# Utilization of Radiographic Imaging for Infant Hydronephrosis over the First 12 Months of Life

**DOI:** 10.1155/2020/2108362

**Published:** 2020-07-30

**Authors:** Anthony J. Schaeffer, Patrick C. Cartwright, Glen A. Lau, Mark D. Ebert, Nora F. Fino, Flory L. Nkoy, Rachel Hess

**Affiliations:** ^1^Division of Urology, Department of Surgery, University of Utah, Salt Lake City, UT, USA; ^2^Intermountain Pediatric Imaging SLC, UT/Department of Radiology, University of Utah, Salt Lake City, UT, USA; ^3^Division of Epidemiology, Department of Internal Medicine, University of Utah, Salt Lake City, UT, USA; ^4^Department of Pediatrics, University of Utah, Salt Lake City, UT, USA; ^5^Division of Health System Innovation and Research, Department of Population Health Sciences, University of Utah, Salt Lake City, UT, USA

## Abstract

**Purpose:**

The workup and surveillance strategies for infant hydronephrosis (HN) vary, although this could be due to grade-dependent differences in imaging intensity. We aimed to describe the frequency of imaging studies for HN within the first year of life, stratified by initial HN grade, within a large regional healthcare system. *Study Design and Data Source*. Retrospective cohort using Intermountain Healthcare Data Warehouse. Inclusion criteria: (1) birth between 1/1/2005 and 12/31/2013, (2) CPT code for HN, and (3) ultrasound (U/S) confirmed HN within four months of birth. *Data Collection*. Grade of HN on initial postnatal U/S; number of HN-associated radiologic studies (renal U/Ss, voiding cystourethrograms (VCUGs), and diuretic renal scans); demographic and medical variables. *Primary Outcome*. Sum of radiologic studies within the first year of life or prior to pyeloplasty. *Statistical Analysis*. Multivariate poisson regression to analyze association between the primary outcome and the initial HN grade.

**Results:**

Of 1,380 subjects (993 males and 387 females), 990 (72%), 230 (17%), and 160 (12%) had mild, moderate, and severe HN, respectively. Compared with those with mild HN, patients with moderate (RR: 1.57; 95% CI: 1.42–1.73) and severe (RR: 2.09; 95% CI: 1.88–2.32) HN had a significantly higher rate of imaging use over 12 months (or prior to surgery) after controlling for potential confounders.

**Conclusions:**

In a large regional healthcare system, imaging use for HN is proportional to its initial grade. This suggests that within our system, clinicians treating this condition are using a risk-stratified approach to imaging.

## 1. Purpose

Antenatal hydronephrosis is found in 1–5% of pregnancies during routine ultrasonographic screening. [1–3] While up to 50% of cases will resolve during gestation, many cases will persist and require postnatal follow-up [4, 5]. Imaging strategies vary for the postnatal workup and surveillance of antenatally diagnosed and postnatally persistent hydronephrosis (PNH) [6–9]. One would expect that the use of imaging would be appropriately higher for those with moderate and severe PNH and more limited for those with mild disease; approximately 50% and 90% of moderate and severe diseases, respectively, are found to have an underlying anatomic abnormality, compared to 12% of those with mild hydronephrosis [10]. Despite attempts to more clearly define the timing and type of imaging postnatally [11], there remains significant variability in the use of imaging both in utero and postnatally [12, 13]. However, the reason for this variability is not well known. Previous work failed to determine whether the variability in use of radiological imaging was due to disease severity, practice variability, or both [12, 13].

The aim of this study is to describe imaging strategies used for the workup and surveillance of PNH, stratified by postnatal ultrasound grade. Specifically, we assessed the frequency of postnatal ultrasounds (U/Ss), voiding cystourethrograms (VCUGs), and diuretic renal scans (DRSs) used within the first year of life among a cohort of patients with PNH. We hypothesized that those with mild hydronephrosis would have less postnatal imaging, whereas those with more severe disease would have more imaging studies performed.

## 2. Materials and Methods

### 2.1. Study Design

This was a retrospective cohort study of children born between 2005 and 2013 with hydronephrosis at Intermountain Healthcare (IH) hospitals.

### 2.2. Setting

IH, a regional not-for-profit integrated health care delivery system in the western United States, has 24 hospitals and 189 outpatient clinics and urgent care facilities serving about 60% of Utah's residents and 85% of Utah's children.

### 2.3. Data Source

After Institutional Review Board approval, we used the IH Enterprise Data Warehouse (EDW) to identify the study cohort and to collect patient information. The EDW is an integrated database that compiles administrative and clinical data from all IH hospitals and clinics; in 2015, the IH EDW captured over 5.7 million unique patients [14]. Inpatient and outpatient hospitalization, pharmacy, radiology, and other encounters are also captured in the EDW, and unique patient identification numbers allow subjects to be followed over time [15]. The IH EDW has been used successfully in a research capacity to identify and follow pediatric patients with a wide range of diseases [16–18].

### 2.4. Patient Identification

The inclusion and exclusion criteria were designed to include patients with isolated nonrefluxing ureteropelvic junction-like hydronephrosis. Specifically, we used the International Classification of Diseases, Ninth Revision, Clinical Modification (ICD-9-CM) codes for hydronephrosis and associated conditions (591*x*, 591.4, 593.5, 753.29, 753.21, 753.1*x*, and 593.7*x*). Subjects were included if they were born between 1/1/2005 and 12/31/2013 and had both the ICD-9 diagnosis code and ultrasound documented hydronephrosis within 4 months of birth. Exclusion criteria included (1) secondary causes for hydronephrosis including urinary stone (592.0, 592.1, 592.9, and 594.1), posterior urethral valves (753.6), spina bifida (756.17), bladder exstrophy (753.5), prune belly syndrome (756.71), congenital megaureters (753.22), or ureteroceles (753.23); (2) isolated vesicoureteral reflux (593.70–3); (3) less than 2 years of follow-up, as defined by an encounter within the EDW at or beyond 24 months of life. The listed follow-up duration was an exclusion criterion because the subjects in this present report are part of a larger study needing 2 years of follow-up.

Demographic characteristics such as gender, prematurity status, race, ethnicity, and insurance status (private/commercial insurance or Medicaid or other governmental insurance) were extracted. Presence and number of non-GU comorbidities was tabulated and classified according to criteria set forth by Fuedtner [19]. ICD-9-CM codes for urinary tract infection (590.0–3, 590.8–9, 595.0, 595.9, and 599.0) were used to determine if a UTI occurred. Finally, as the frequency of imaging may be determined by proximity to a major urban center, we used the U.S. Office of Management and Budget's Core-Based Statistical Areas to determine whether the patient resided in an urban, rural, or frontier location (frontier and rural locations combined) [20]. The numbers of renal ultrasounds, voiding cystourethrograms (VCUGs), diuretic nuclear medicine renal scans (DRS), and other radiologic imaging tests of the urinary tract were tabulated within the first year of life, as was a surgery to correct a ureteropelvic junction obstruction (i.e., pyeloplasty).

Typical endpoints for ultrasound follow-up, if it occurred, varied based on hydronephrosis grade. For mild hydronephrosis, the endpoint for ultrasound follow-up was either improvement to no hydronephrosis or stability of mild hydronephrosis visualized on at least two sequential ultrasounds. For moderate cases, ultrasound follow-up ceased with improvement to mild hydronephrosis or stability of moderate hydronephrosis visualized on at least two sequential ultrasounds without any loss in renal function as measured by DRS. For severe cases, ultrasound follow-up was complete if there was improvement to moderate or mild hydronephrosis visualized on at least two sequential ultrasounds or stability of severe hydronephrosis without any loss in renal function as measured by DRS. Typical indications for follow-up DRS were persistent or worsening severe and/or worsening moderate hydronephrosis. The frequency of DRS during follow-up was not standardized, was clinician dependent, and thus varied between 3 and 12 months. These are general imaging indications and endpoints and may have differed on a case-by-case basis.

If surgery occurred prior to the first birthday, subjects were censored on the day of surgery; imaging use up to surgery was included for analysis. The indications for pyeloplasty typically included an initial differential renal function of <40% measured by DRS, >10% worsening in renal function as measured by DRS, worsening drainage (i.e., increase in slope of the drainage curve and/or longer half-time) as measured by DRS, and/or worsening ultrasound appearance (i.e., increase in swelling of calyces or pelvis or even less thickness of renal parenchyma) of the hydronephrotic kidney. The last three indications were in comparison to prior DRS or ultrasound, where appropriate.

### 2.5. Statistical Analysis

The unit of analysis is the subject's radiology report. Hydronephrosis was confirmed by manually abstracting and assigning grades of hydronephrosis (mild, moderate, and severe categories) from the reports. The mild, moderate, and severe grading categories were used because the Society for Fetal Urology (SFU) grading system was published immediately before the study period began and was not implemented throughout the IH system until more recently [21]. The mild category includes SFU grade 1 and grade 2, the moderate category includes SFU grade 3, and the severe category includes SFU grade 4. As the studies could have been performed at any IH facility, radiologists with varying familiarity with pediatric uroradiology and from different practice settings throughout the IH system performed and interpreted the studies. Therefore, agreement between the hydronephrosis grade on the radiology report and the grade assigned after ultrasound imaging review by a fellowship trained pediatric urologist (GAL and AJS) was calculated for 139 randomly selected patient images and reports using Cohen's kappa statistic.

The primary outcome for this study was the sum of radiologic studies each patient received within the first year or prior to surgery. Differences in demographic characteristics were assessed using chi-squared tests. Poisson regression was used to analyze the association between the primary outcome and the initial grade of PNH, controlling for gender, prematurity, race/ethnicity, insurance type, presence of comorbidity, presence of UTI, and urban versus rural residence. Similar models were performed for the number of ultrasounds (without including diuretic renal scans and VCUGs). All models included the log length of the observation period (either 12 months or time until surgery) as an offset. Poisson assumptions were checked and verified. We calculated rate ratios (RR) and corresponding 95% confidence intervals (CI) from models. Almost all fluoroscopic and nuclear medicine studies for patients in this region are performed at an IH facility (and captured by the EDW) regardless of insurance status. However, patients with insurance coverage outside of the IH system may have been more likely to have studies that were not captured by the data source. Therefore, we performed a sensitivity analysis comparing the use of imaging amongst those with and without an IH-based insurance plan. All analyses were done in SAS version 9.4 (Cary, NC). A two-tailed *p* < 0.05 was considered statistically significant.

## 3. Results

The cohort consisted of 1,380 subjects (993 males and 387 females), of which 990 (72%), 230 (17%), and 160 (12%) had mild, moderate, and severe hydronephrosis, respectively (Table 1). The interrater reliability between the interpreting radiologist and the pediatric urologist for hydronephrosis grade on ultrasound was 0.82. The majority of patients were male (72%), white (73%), and lived in an urban (93%) location. In the first year of life or before surgery, 2,529 ultrasounds, 597 diuretic renal scans, and 838 VCUGs were performed. For those with mild hydronephrosis on their first ultrasound, 76% remained mild at 1 year, whereas 17% and 7% progressed to moderate and severe hydronephrosis, respectively. For those with moderate hydronephrosis on their index ultrasound, 25% remained moderate at one year, 45% improved to mild, and 30% worsened to severe hydronephrosis. For those with severe hydronephrosis at birth, 79% remained severe at 1 year, 8% improved to moderate hydronephrosis, and 13% improved to mild. The median [IQR] of ultrasounds performed in the first 12 months of life for mild hydronephrosis was 2 [1, 2], whereas for moderate and severe hydronephrosis the medians were 2 [1, 2] and 2 [1, 3], respectively. The maximum number of ultrasounds performed in the first year or before surgery were 5, 6, and 5 for mild, moderate, and severe hydronephrosis, respectively. The use of VCUG and renal scans for each patient increased with the severity of hydronephrosis (Table 1). Eight percent and 36% of patients with moderate and severe hydronephrosis in their initial ultrasound, respectively, underwent a pyeloplasty within the first year of life, as compared to <1% of those with mild hydronephrosis on their initial ultrasound. When imaging use is taken as a composite, the results demonstrate that there is more imaging for more severe grades of hydronephrosis after controlling for potential confounders on multivariate analysis (Table 2). Specifically, compared to patients with mild hydronephrosis, patients with moderate (RR: 1.57; 95% CI: 1.42–1.73) and severe (RR: 2.09; 95% CI: 1.88–2.32) hydronephrosis had a significantly higher rate of imaging use over 12 months (or prior to surgery). When considering only ultrasound use, the results are consistent with those of the primary outcome. Specifically, compared with patients with mild hydronephrosis, more ultrasounds were performed among patients with moderate (RR: 1.28; 95% CI: 1.13–1.45) and severe (RR: 1.45; 95% CI: 1.25–1.68) hydronephrosis (data not shown).

Patients living in a rural Utah location were less likely to receive imaging compared with those in an urban location (RR 0.81; 95% CI: 0.68–0.97). Also, we found no difference in imaging use between the patients with and without Intermountain Healthcare insurance plan coverage.

## 4. Discussion

Despite consensus statements by pediatric nephrologists, radiologists, urologists, and obstetricians that attempted to standardize the follow-up, medical management and imaging selections for PNH [4, 11], many different imaging strategies are still used [6, 7, 9, 22]. This leads to the potential for significant variability in use of imaging over the first year of life. An in-depth analysis of imaging strategies used in patients with PNH is critically needed to uncover reasons for practice variation and guide the development of clearer guidelines for optimal resource allocation.

In this study, we used data from a large regional database to measure the use of imaging over the first year of life. This analysis was unique in that we were able perform analysis stratified by the initial severity of hydronephrosis and control for other factors that could influence imaging. We found that the use of postnatal imaging within the first year of life or prior to surgery to correct a ureteropelvic junction obstruction is proportional to the initial ultrasound grade of hydronephrosis. That is, the number of imaging studies increases modestly with the grade of hydronephrosis. These results differ from other studies, which showed wider variability in the use of ultrasound and other imaging studies for patients with hydronephrosis. Dy and colleagues used MarketScan, an administrative database of commercially insured patients, to study prenatal and postnatal resource utilization in PNH, including ultrasound use. [23] These authors excluded patients who underwent surgery in infancy, thereby limiting the cohort to patients with milder disease. While there are some similarities comparing their entire cohort (which mostly represented mild disease) to our mild group in the number of average (about 2 for both studies) and maximum (5 in the present study versus 7 in the Dy study), 30% of patients in their cohort had 3 or more ultrasounds compared with 16% of mild PNH patients in this present study. The difference could be due to the sample size differences, changes in ultrasound grade, different catchment areas, or variable geographic practice patterns. Regardless of reason for the difference, the economic implications and potential cost savings of reducing imaging for milder disease are substantial.

Another interesting finding in our study that merits further inquiry is the lower imaging rate we identified among patients living in a rural setting in Utah. Rural-urban disparities in access to subspecialty and intensive care as well as disease prevalence in pediatric medicine are well recognized [24–27]. A recent study demonstrated that access to routine pediatric urology imaging studies such as VCUG and diuretic renal scans were seldom offered at rural hospitals within Washington state [28]. The same access difficulties are present within the IH system, with much of specialized uroradiographic testing occurring at the only freestanding tertiary care pediatric hospital in the Intermountain West. Telehealth attempts to improve access and streamline care for rural patients in the IH and other systems [29, 30]. However, the most important question, and one that is the subject of ongoing work by our group, is whether the reduced imaging rate leads to worse long-term renal function.

This study should be viewed in light of its limitations. First, our analysis was unable to account for changing grades of ultrasounds within the study period which could have affected imaging frequency. This was due to the relatively short timeframe and small absolute number of cases that changed from their initial grade. However, we anticipate that the differences seen in imaging strategy would be even greater if the 25% of moderate and 13% of severe initial ultrasound grades that improved to mild were taken into account. Although the database allowed for grading of ultrasounds and captured patients within one healthcare system, it is possible that some radiographic studies could be missed if they were performed elsewhere. However, our sensitivity analysis, which assumes that IH insured patients would obtain all of their radiology studies at IH facilities, showed that there was no difference in imaging use between those with Intermountain insurance and those without. Also, we expect that nearly all nuclear medicine and the majority of fluoroscopic studies would be performed at IH's tertiary care pediatric hospital, yet some may have been performed at other nonspecialized unaffiliated radiology centers or even outside our state. Even if we missed studies, we would expect that they would be on the more severe cases, which would further support our findings of increased imaging for more severe diseases.

## 5. Conclusions

Our results show that, in a large regional healthcare system, imaging use postnatally confirmed hydronephrosis is proportional to the initial grade of hydronephrosis. This suggests that, within our large system, clinicians treating this condition are using a rational and risk-stratified approach to imaging.

## Figures and Tables

**Table 1:** Demographics of infant hydronephrosis cohort.

Variable	Total cohort (*n* = 1,380)	Initial hydronephrosis grade			*p* value
		Mild (*n* = 990)	Moderate (*n* = 230)	Severe (*n* = 160)	
	*n* (%)	*n* (%)	*n* (%)	*n* (%)	
Delivery					<0.001
Premature	173 (18.6)	148 (21.7)	15 (10.4)	10 (9.8)	
Term	756 (81.4)	535 (78.3)	129 (89.6)	92 (90.2)	

Gender					0.57
Male	993 (72.0)	706 (71.3)	172 (74.8)	115 (71.9)	
Female	387 (28.0)	284 (28.7)	58 (25.2)	45 (28.1)	

Race/ethnicity					0.07
White	1008 (73.0)	706 (71.3)	171 (74.3)	131 (81.9)	
Hispanic	261 (18.9)	202 (20.4)	39 (17.0)	20 (12.5)	
Others	111 (8.0)	82 (8.3)	20 (8.7)	9 (5.6)	

Insurance status					0.78
Private/commercial	872 (63.2)	623 (62.9)	142 (61.7)	107 (66.9)	
Medicaid	461 (33.4)	335 (33.8)	78 (33.9)	48 (30.0)	
Public employee/military	47 (3.4)	32 (3.2)	10 (4.3)	5 (3.1)	

Residence					0.24
Urban	1289 (93.4)	930 (93.9)	209 (90.9)	150 (93.8)	
Rural	91 (6.6)	60 (6.1)	21 (9.1)	10 (6.3)	

No. of non-GU comorbidities					<0.001
0	1077 (78.0)	783 (79.1)	177 (77.0)	117 (73.1)	
1	161 (11.7)	90 (9.1)	39 (17.0)	32 (20.0)	
2	56 (4.1)	39 (3.9)	9 (3.9)	8 (5.0)	
3+	86 (6.2)	78 (7.9)	5 (2.2)	3 (1.9)	

Infant UTI					0.96
No	1234 (89.4)	886 (89.5)	206 (89.6)	142 (88.8)	
Yes	146 (10.6)	104 (10.5)	24 (10.4)	18 (11.3)	

No. of CT and MRIs					0.27
None	1375 (99.6)	988 (99.8)	228 (99.1)	159 (99.4)	
1 or more	5 (0.4)	2 (0.2)	2 (0.9)	1 (0.6)	

No. of diuretic renal scans					<0.001
None	897 (65.0)	798 (80.6)	84 (36.5)	15 (9.4)	
1	372 (27.0)	171 (17.3)	110 (47.8)	91 (56.9)	
2 or more	111 (8.0)	21 (2.1)	36 (15.7)	54 (33.8)	

No. of VCUGs					<0.001
None	556 (40.3)	505 (51.0)	43 (18.7)	8 (5.0)	
1	810 (58.7)	476 (48.1)	185 (80.4)	149 (93.1)	
2 or more	14 (1.0)	9 (0.9)	2 (0.9)	3 (1.9)	

No. of renal ultrasounds					<0.001
1	554 (40.1)	437 (44.1)	63 (27.4)	54 (33.8)	
2	575 (41.7)	399 (40.3)	111 (48.3)	65 (40.6)	
3 or more	251 (18.2)	154 (15.6)	56 (24.3)	41 (25.6)	
Median (IQR)		2 [1, 2]	2 [1, 2]	2 [1, 3]	
Max		5	6	5	

Infant pyeloplasty					<0.001
No	1302 (94.3)	987 (99.7)	212 (92.2)	103 (64.4)	
Yes	78 (5.7)	3 (0.3)	18 (7.8)	57 (35.6)	

Treated at tertiary care facility					0.77
Yes	937 (83.6)	675 (83.4)	154 (82.8)	108 (85.7)	
No	184 (16.4)	134 (16.6)	32 (17.2)	18 (14.3)	

**Table 2:** Association between total imaging use in infancy and initial postnatal ultrasound grade of hydronephrosis.

	Unadjusted		Adjusted	
	Rate ratio (95% CI)	*p* value	Rate ratio (95% CI)	*p* value
Initial hydronephrosis grade				
Mild	Ref		Ref	
Moderate	1.55 (1.44–1.68)	<0.001	1.57 (1.42–1.73)	<0.001
Severe	2.15 (1.97–2.34)	<0.001	2.09 (1.88–2.32)	<0.001

Delivery				
Premature	0.94 (0.85–1.03)	0.19	1.07 (0.96–1.19)	0.23
Term	Ref		Ref	

Gender				
Male	1.02 (0.95–1.09)	0.62	1.02 (0.94–1.11)	0.63
Female	Ref		Ref	

Race/ethnicity				
Hispanic	0.99 (0.91–1.07)	0.74	1.08 (0.96–1.20)	0.21
White	Ref		Ref	
Others	0.97 (0.86–1.09)	0.58	1.02 (0.87–1.19)	0.83

Insurance status				
Private/commercial	Ref		Ref	
Medicaid	0.96 (0.90–1.03)	0.25	0.93 (0.85–1.02)	0.15
Public employee/military	1.11 (0.95–1.31)	0.19	1.04 (0.86–1.27)	0.67

Residence				
Rural	0.91 (0.80–1.04)	0.18	0.81 (0.68–0.97)	0.02
Urban	Ref		Ref	

No. of non-GU comorbidities				
0	Ref		Ref	
1	1.18 (1.07–1.29)	0.001	1.07 (0.96–1.19)	0.25
2	1.03 (0.88–1.21)	0.71	0.99 (0.82–1.20)	0.93
3+	0.79 (0.69–0.91)	0.001	0.86 (0.73–1.01)	0.06

## Data Availability

The data supporting the findings of this study are available from the corresponding author upon request.
